# Accelerated recovery from facial paralysis using individual‐target transcranial magnetic stimulation after masseteric–facial nerve end‐to‐end anastomosis: A case report

**DOI:** 10.1111/cns.14084

**Published:** 2023-01-10

**Authors:** Shun Qi, Yang Rao, Chuanzhu Sun, Zhou Fei, Sanzhong Li

**Affiliations:** ^1^ The Key Laboratory of Biomedical Information Engineering of Ministry of Education Institute of Health and Rehabilitation Science School of Life Science and Technology The Key Laboratory of Neuro‐informatics & Rehabilitation Engineering of Ministry of Civil Affairs Xi'an Jiaotong University Xi'an China; ^2^ Shaanxi Brain Modulation and Scientific Research Center Xi'an China; ^3^ Department of Neurosurgery The 1st Affiliated Hospital of Air Force Medical University Xi'an China

## 1 INTRODUCTION

Dear Editor,

The resection of intracranial tumors, such as acoustic neuroma, often causes facial paralysis.^1^ Injury to the facial nerve is the immediate cause of this paralysis. Nerve anastomosis, including hypoglossal–facial^2^ and masseteric–facial nerve anastomosis (MFA),^3^ is an effective method to remodel the injured nerve and establish a nerve regeneration pathway.^4^, ^5^ However, regaining normal facial function and good symmetry takes more than 12 months.^6^ Repetitive transcranial magnetic stimulation (rTMS) applies a continuously adjustable magnetic field to the central nervous system (CNS), achieving cumulative curative results. However, whether rTMS can be used to promote recovery from facial paralysis is known. Here, we report accelerated facial recovery with a novel form of rTMS, individual‐target TMS (IT‐TMS). The patient's recovery period was reduced to approximately 6 months with a corresponding improvement in House–Brackmann (HB) scale results.

## 2 CASE REPORT

**FIGURE 1:**
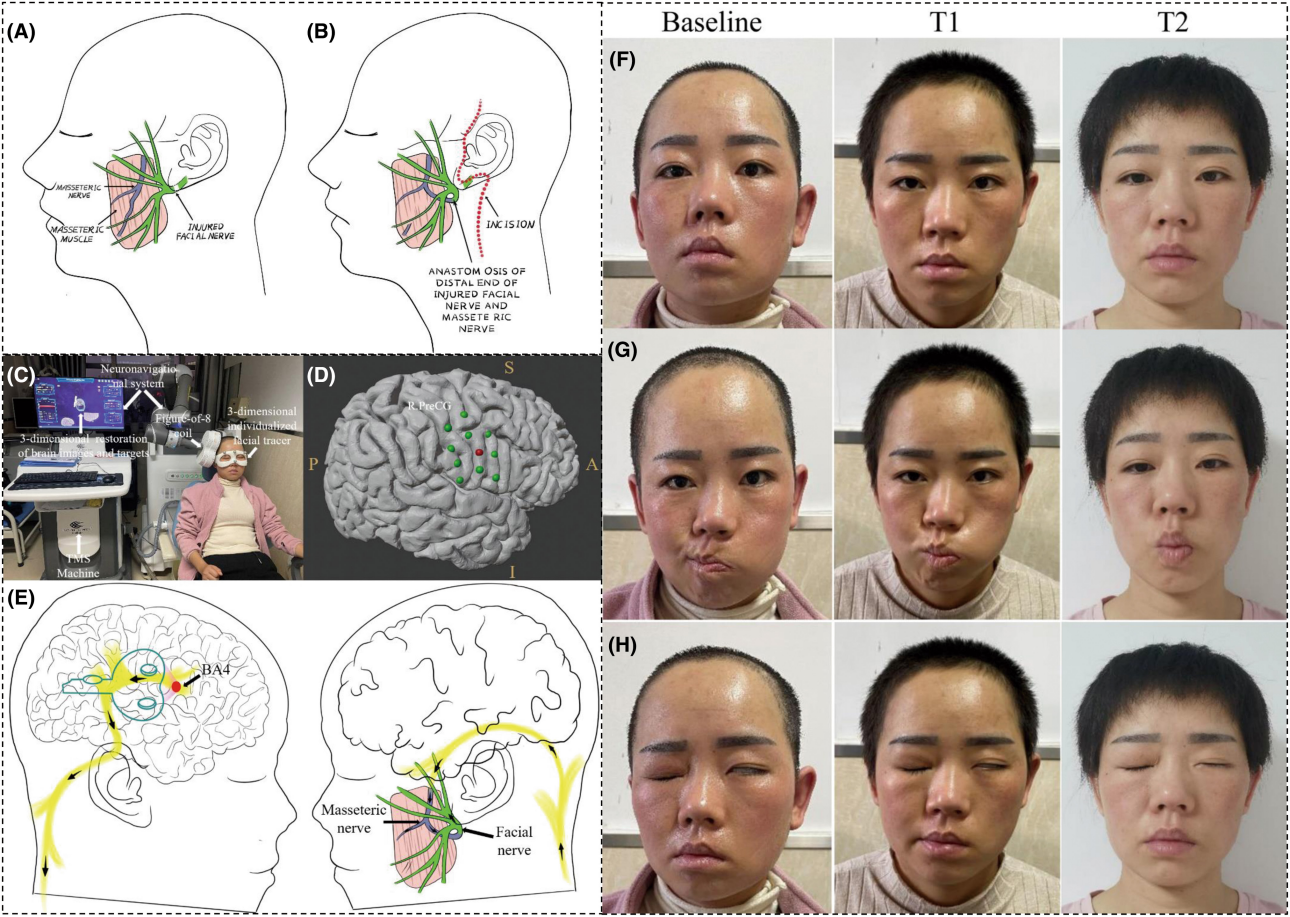
Drawing of masseteric–facial nerve anastomosis. (A) The relative anatomical positions of the facial nerve and the masseteric nerve anatomically on the paralyzed side were identified. (B) The masseteric nerve was cut off from one of the distal ends and anastomosed with the main trunk of the extracranial facial nerve. (C) The IT‐TMS experimental set‐up is shown with labeled devices. (D) Green dots indicate the representative sites corresponding to masseteric muscles, and the masseteric hotspot is shown in red. (E) The schematic diagram shows the signal evoked by the stimulation coil proceeding from the masseteric muscle target (red dot) located in the precentral gyrus of Brodmann 4 area to masseteric nerve through fasciculi (yellow tracts), ultimately arriving at the facial nerve. Static (F), dynamic (G), and eye closure (H) images of the face at baseline and at 1 month (T1) and 6 months (T2) after the initiation of IT‐TMS. S: superior; I: inferior; A: anterior; P: posterior; R.PreCG: right precentral gyrus; BA4: Brodmann 4 area.

The patient was a 33‐year‐old woman who had surgery for the removal of a left acoustic neuroma on June 15, 2021. Then she gradually developed facial paralysis on the left side, evaluated as grade VI using the HB scale. The paralysis was initially complicated by loss of control of the left facial muscles, deviation of the mouth to the right side, facial asymmetry, and incomplete eyelid closure. Symptoms progressed to left facial dystonia and stiffness, loss of control of the angulus oris on the lesioned side, an expanded area of unclosed eyelid, and self‐reported insomnia. Four months later, the patient underwent MFA (Figure 1A,B) on October 16, 2021. During the following 1 month prior to IT‐TMS treatment, the patient received daily traditional Chinese medicine treatment. She was also prescribed nerve growth factor and vitamin B12. However, her facial function did not improve and the facial grade VI persisted. The patient denied a history of facial trauma or other tumor surgery, and she had had a normal facial function before the removal of the acoustic neuroma. The possibility of epilepsy, magnetic resonance imaging (MRI) contraindications, and other diseases were excluded (Table S1). The patient provided written informed consent. This study was approved by the Ethics Committee of Xijing Hospital and conducted under the Declaration of Helsinki.

IT‐TMS (Figure 1C) was initiated at the Department of Neurosurgery, Xijing Hospital, on November 15, 2021. IT‐TMS was performed under established safety guidelines and protocols,^7^ using a stimulator with a figure‐8 coil and a neuronavigational system, the Black Dolphin Navigation Robot (S‐50, Solide Company, Xi'an, China), with a coupled three‐dimensional individualized facial tracer and infrared cameras that guided the precise and repetitive targeting over the same site across different sessions. The specific anatomical sites are in the precentral gyrus that are corresponding to masseteric muscles (Figure 1D). Briefly, the predefined targets, based on individual MRI brain images collected prior to treatment, were integrated into the operation system, in which a three‐dimensional restoration of brain images and targets was visualized (Figure 1C). This process allowed the visual operation for target selection and monitoring. The patient received 10 Hz stimulation at 120% of the resting motor threshold delivering 18 trains with an inter‐train interval of 8 s (1800 pulses per session, 2 sessions/3600 pulses per day, 5 days/10 sessions per week, 1 week interval after 1 week stimulation). The intersession interval was 50 min, and the treatment lasted 6 months. The schematic signal transduction evoked by IT‐TMS is shown in Figure 1E.

The patient was instructed by a professional physiotherapist to perform 30 min of facial exercises daily (as shown in Video S1 and S2) immediately after IT‐TMS. She was also encouraged to do the daily exercises during the weekly interval as well.

The clinical information is summarized in Table S1. The HB results are shown in Table 1 for baseline, timepoint 1 (T1), and timepoint 2 (T2). The records for three facial actions are shown. Before IT‐TMS treatment (baseline), the patient had obvious facial asymmetry at rest (Figure 1F), a disfigured face when attempting to use mimetic musculature (Figure 1G), and a broad eyelid gap (Figure 1H). After the first month of treatment (T1), her symptoms were partially improved, as indicated by less asymmetry, oral improvement, and more closure of the eyelid. Six months later (T2), the patient had complete facial symmetry, restored facial expression, and almost complete closure of the eyelid. The movement ability and stiffness were significantly improved after 6 months of IT‐TMS treatment. The patient also reported good sleep and recovery from dry eyes. Her treatment was terminated on May 15, 2022. No significant adverse effects were reported during or after treatment.

**TABLE 1:** The results of modified House–Brackmann facial grading scale

Time point	Modified House–Brackmann facial grading scale system					
	Score				Total score	Grade
	Eyebrow	Eye	Nasolabial fold	Oral		
Baseline	6	6	6	6	24	VI
T1	6	5	6	5	22	V
T2	2	2	1	1	6	II

Abbreviations: Baseline, before IT‐TMS treatment; T1, after the first 1‐month treatment; T2, 6 months later.

## 3 DISCUSSION

Patients with facial paralysis have been reported to have a reduced area of the brain cortex corresponding to facial function on the lesioned side, and the remodeling of facial representations is closely correlated with a better prognosis.^8^ In our study, IT‐TMS targeting masseteric muscle representations successfully improved facial stiffness, strength, and symmetry. Compared with previously published results,^6^ the addition of IT‐TMS shortened the time needed for rehabilitation, from more than 12 months to approximately 6 months. The significantly accelerated process allowed better recovery within a shorter period of time and allowed the patient a quicker return to normal life. The effects could be attributed to the projections of neural impulses activated by IT‐TMS from the CNS into the facial nerve (Figure 1E), helping to strengthen masseteric–facial nerve transfer and remodel cortical representations. IT‐TMS, combined with facial exercises, led to excitatory after‐effects and improved recovery. Moreover, IT‐TMS guaranteed the precise stimulation over the masseteric hotspot and therefore offered significant efficacy, notably improving the clinical treatment of facial paralysis.

In conclusion, IT‐TMS could be a promising means of neurostimulation to accelerate facial recovery for patients who would otherwise undergo a longer period of rehabilitation. A larger‐sample trial is going on to verify the effects demonstrated in this case study and to optimize the parameters. In addition, it would be worthwhile to study whether a higher dose of stimulation could lead to a shorter recovery period. Comparison with a sham group and an exercise‐only group are also warranted.

## 4 FUNDING INFORMATION

This work was supported by Academic Discipline Boosting Program of Xijing Hospital (grant number XJZT19Ml13).

## 5 CONFLICT OF INTEREST

The authors declare that they have no conflict of interest.

6

## Supporting information


Facial exercises‐facial movement

Facial exercises‐muscle massage

Supplementary Table 1‐An overview of clinical information of this patient

## Data Availability

The data that support the findings of this study are available from the corresponding author upon reasonable request.
