# Supplementary material for: Accelerated recovery from facial paralysis using individual‐target transcranial magnetic stimulation after masseteric–facial nerve end‐to‐end anastomosis: A case report
Source: CNS Neurosci Ther. 2023 Jan 10;29(4):1197–9. doi: 10.1111/cns.14084 (PMC10018098; doi:10.1111/cns.14084)
Supplement: Supplementary file 3 — Supplementary Table 1‐An overview of clinical information of this patient [file CNS-29-1197-s001.docx]

SUPPLEMENTARY TABLE 1

An overview of clinical information of this patient.

| Examination | Results |
| --- | --- |
| Age | 33 |
| Sex | Female |
| Time of acoustic neuroma ectomy | June 15, 2021 |
| Time of MFA | October 16, 2021 |
| Initiation of IT-TMS treatment | November 15, 2021 |
| House-Brackmann facial grading | Ⅵ, facial paralysis |
| Physical examination | Left side: facial stiffness and asymmetry, lost control of angulus oris, unclosed eyelid |
| Internal examination | Normal |
| Neurological examination | Injury of facial nerve root that projects into facial muscles on left side |
| Psychiatric examination | Normal |
| Brain MRI | DTI: Disruption of nerve fiber tracts that projects into facial muscles on left side |
| Blood test | Normal |
| Stool test | Normal |
| Urine test | Normal |
| IQ test | Normal |

Abbreviations: MFA, masseteric-facial nerve anastomosis; IT-TMS, individual-target transcranial magnetic stimulation; MRI, magnetic resonance imaging; DTI, diffusion tensor imaging; IQ, intelligence quotient
